# SPG20 Protein Spartin Associates with Cardiolipin via Its Plant-Related Senescence Domain and Regulates Mitochondrial Ca^2+^ Homeostasis

**DOI:** 10.1371/journal.pone.0019290

**Published:** 2011-04-29

**Authors:** Dinesh C. Joshi, Joanna C. Bakowska

**Affiliations:** Department of Molecular Pharmacology and Therapeutics, Loyola University Chicago, Maywood, Illinois, United States of America; National Institutes of Health, United States of America

## Abstract

Hereditary spastic paraplegias (HSPs) are a group of neurological disorders characterized clinically by spasticity of lower limbs and pathologically by degeneration of the corticospinal tract. Troyer syndrome is an autosomal recessive HSP caused by a frameshift mutation in the spartin (SPG20) gene. Previously, we established that this mutation results in a lack of expression of the truncated mutant spartin protein. Spartin is involved in many cellular processes and associates with several intracellular organelles, including mitochondria. Spartin contains a conserved plant-related senescence domain at its C-terminus. However, neither the function of this domain nor the roles of spartin in mitochondrial physiology are currently known. In this study, we determined that the plant-related senescence domain of spartin interacts with cardiolipin but not with two other major mitochondrial phospholipids, phosphatidylcholine and phosphatidylethanolamine. We also found that knockdown of spartin by small interfering RNA in a human neuroblastoma cell line resulted in depolarization of the mitochondrial membrane. In addition, depletion of spartin resulted in a significant decrease in both mitochondrial calcium uptake and mitochondrial membrane potential in cells treated with thapsigargin. Our results suggest that impairment of mitochondrial calcium uptake might contribute to the neurodegeneration of long corticospinal axons and the pathophysiology of Troyer syndrome.

## Introduction

The hereditary spastic paraplegias (HSPs) are inherited neurological disorders characterized by a common feature of progressive spasticity in the lower limbs with degeneration of corticospinal projections of motor neurons [1]. Troyer syndrome (SPG20) is an autosomal recessive HSP, in which patients show spasticity of lower limbs as well as other symptoms, including mental retardation, dysarthria, and short stature [2]. The disease is caused by a frameshift mutation in the spartin gene (SPG20) [3] resulting in a lack of expression of spartin rather than expression of a truncated protein [4], indicating that the pathogenesis of Troyer syndrome results from a loss-of-function mechanism.

Spartin harbors two conserved domains, an MIT (microtubule interacting and trafficking motif) domain at the N-terminus and a plant-related senescence domain at the C-terminus [5]. Currently, neither the function nor the binding partners of the plant-related senescence domain are known. The following evidence suggests that the spartin protein plays diverse roles in the biology the cell: the presence of different structural domains within spartin [5], its association with several intracellular organelles [6]–[9] and its interaction with many binding partners [10], [11]. Thus far, spartin is known to play a role in the trafficking of the epidermal growth factor receptor [7], [8] and in the turnover of lipid droplets [12], [13]. Both overexpressed and endogenous spartin have been found to associate with endosomes [7], [8], lipid droplets [8], [12], and mitochondria [6]. However, the localization of spartin in the mitochondria is controversial; an earlier study showed that overexpressed spartin associates with mitochondria via its C-terminus [6], but studies by Eastman and colleagues did not confirm those findings [12].

Mitochondria are key organelles that are critical for generating adenosine triphosphatase (ATP) via oxidative phosphorylation; they are also involved in regulating intracellular Ca^2+^ levels and generating reactive oxygen species (ROS). Impaired mitochondrial function is implicated in the pathogenesis of several neurodegenerative diseases, including Huntington's disease [14], amyotrophic lateral sclerosis [15], as well as HSP7 [16] and HSP13 [17].

HSP7 is caused by a mutation in the paraplegin gene encoding the AAA (ATPases associated with diverse cellular activities) protease located in the inner mitochondrial membrane [18]. Paraplegin protein participates in the degradation of misfolded proteins in the mitochondrial intermembrane space and is important for the assembly of respiratory complexes [19]. Fibroblasts derived from HSP7 patients are more prone to oxidative stress and show impaired activity of mitochondrial complex I compared with fibroblasts derived from unaffected individuals [19].

HSP13 is due to a mutation in the gene encoding heat-shock protein 60 (Hsp60) [17], a chaperonin involved in the folding of proteins that translocate from the cytoplasm to the mitochondrial matrix. It has been shown that decreased levels of Hsp60 activity result in increased cell death and sensitivity to oxidative stress [20].

Currently how spartin associates with the mitochondria and its potential role in mitochondrial functions are not known. In this study we determined that endogenous spartin is localized to mitochondria. Furthermore, we discovered that spartin, via its plant-related senescence domain, associates with cardiolipin, a major mitochondrial phospholipid. We found that cells depleted of spartin and neurons derived from *Spg20* knock-out (KO) mice have depolarized mitochondrial membrane (ΔΨm). In addition, treatment of spartin-depleted cells with thapsigargin, which increases the cytosolic calcium levels, resulted in decreased capacity of mitochondrial calcium uptake and depolarization of the mitochondrial membrane.

## Results

### Endogenous spartin localizes to mitochondria

**Figure 1:**
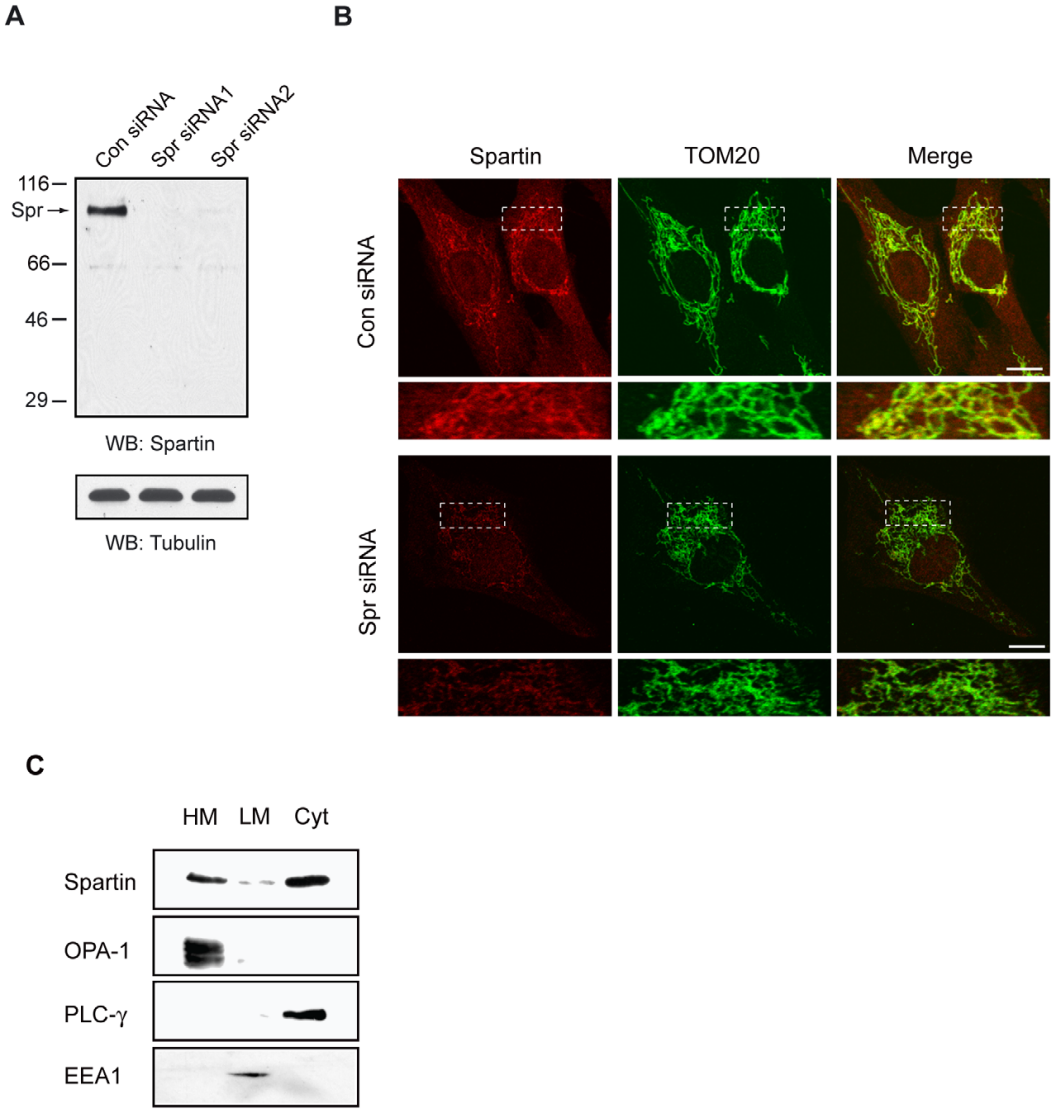
Endogenous spartin associates with mitochondria. (**A**) Detection of endogenous spartin protein in SK-N-SH cells treated with control siRNA, spartin siRNA1, and spartin siRNA2. Cell lysates were immunoblotted with guinea pig polyclonal anti-spartin antibodies. The arrow identifies an ∼85 kD spartin protein. Even loading was confirmed by immunoblotting with anti-tubulin antibodies. Sizes of protein standards are indicated to the left in kDa. (**B**) Localization of endogenous spartin to mitochondria by immunofluorescence. SK-N-SH cells were treated with control (upper panels) or spartin siRNA (lower panels), fixed, and immunostained with anti-spartin (red) and anti-TOM20 (green) antibodies. Merged images are shown in the far right panels. The boxed areas are enlarged and placed below. Scale bar = 10 µm. (**C**) Cells were fractionated using differential centrifugation. Mitochondria-enriched heavy-membrane fraction (HM), light-membrane fraction (LM), and cytosolic fraction (Cyt) were prepared as described in Materials and Methods. 30 µg of protein from each fraction was loaded per lane and analyzed by immunoblotting using anti-spartin, anti-OPA1 (mitochondrial marker), anti-PLC-γ (cytoplasmic marker), or anti-EEA1 (endosomal marker) antibodies.

We examined the subcellular localization of endogenous spartin in the SK-N-SH neuroblastoma cell line by immunofluorescence using a recently developed polyclonal antibody against spartin. First, we examined the specificity of polyclonal antibodies against human spartin by immunoblotting using cell lysates from SK-N-SH cells treated with control or spartin siRNA1 and siRNA2. Endogenous spartin was detected as a doublet: immunoblotting revealed a major, strong band at ∼85 kDa and a much weaker, slower migrating band at ∼95 kDa when cells were treated with control siRNA (Figure 1A). Both bands represent endogenous spartin because neither of them was detected when cells were treated with spartin siRNA1 or siRNA2 (Figure 1A). These results are in agreement with previously published data demonstrating that the fast migrating band corresponds to spartin protein, whereas the slow migrating band corresponds to a mono-ubiquitinated species of spartin [7], [8].

Then, we applied these anti-spartin antibodies in an immunofluorescence assay. As shown in Figure 1B, confocal microscopy analysis of SK-N-SH cells (treated with control siRNA) revealed the immunofluorescence staining of spartin in the mitochondria as demonstrated by colocalization of spartin's signal with TOM20, a mitochondrial marker. Spartin staining was also observed within the cytoplasm. A similar staining pattern of endogenous spartin was observed in primary human fibroblasts, myoblasts, astrocytes, and HeLa cells (data not shown). Importantly, when SK-N-SH cells were treated with spartin siRNA nearly no immunofluorescence staining of spartin was observed, which verifies the specificity of the antibody and spartin's localization to the mitochondria (Figure 1C).

To confirm the expression of spartin in the cytoplasm and mitochondria, we examined the subcellular localization of endogenous spartin by biochemical assays. Specifically, we used differential centrifugation to separate cell homogenates into the heavy-membrane (enriched in the mitochondria), light-membrane (enriched in endosomes), and cytosolic fractions with subsequent analysis by immunoblotting. Most endogenous spartin localized to the cytosol, but a portion of it partitioned to the mitochondria-enriched heavy-membrane fraction, as evidenced by the presence of a mitochondrial marker, OPA-1 and a lack of the cytosolic marker PLC-γ, in this fraction (Figure 1C). A small fraction of endogenous spartin also localized to light-membrane (enriched in endosomes) as demonstrated by the presence of EEA1 marker in this fraction (Figure 1C).

### The plant-related senescence domain of spartin binds to cardiolipin

It has been shown by immunofluorescence that the C-terminus of spartin is responsible for its association with the mitochondria [6]. We confirmed these results using differential fractionation of cell homogenates transfected with HA-spartin (1–408) or HA-spartin (409–666). Immunoblotting revealed that the entire post-nuclear pool of HA-spartin (409–666) that encompasses the C-terminus of spartin was detected in the heavy-membrane fraction containing mitochondria (Figure S1). In contrast, HA-spartin (1–408) was detected exclusively in the cytosolic fraction (Figure S1).

**Figure 2:**
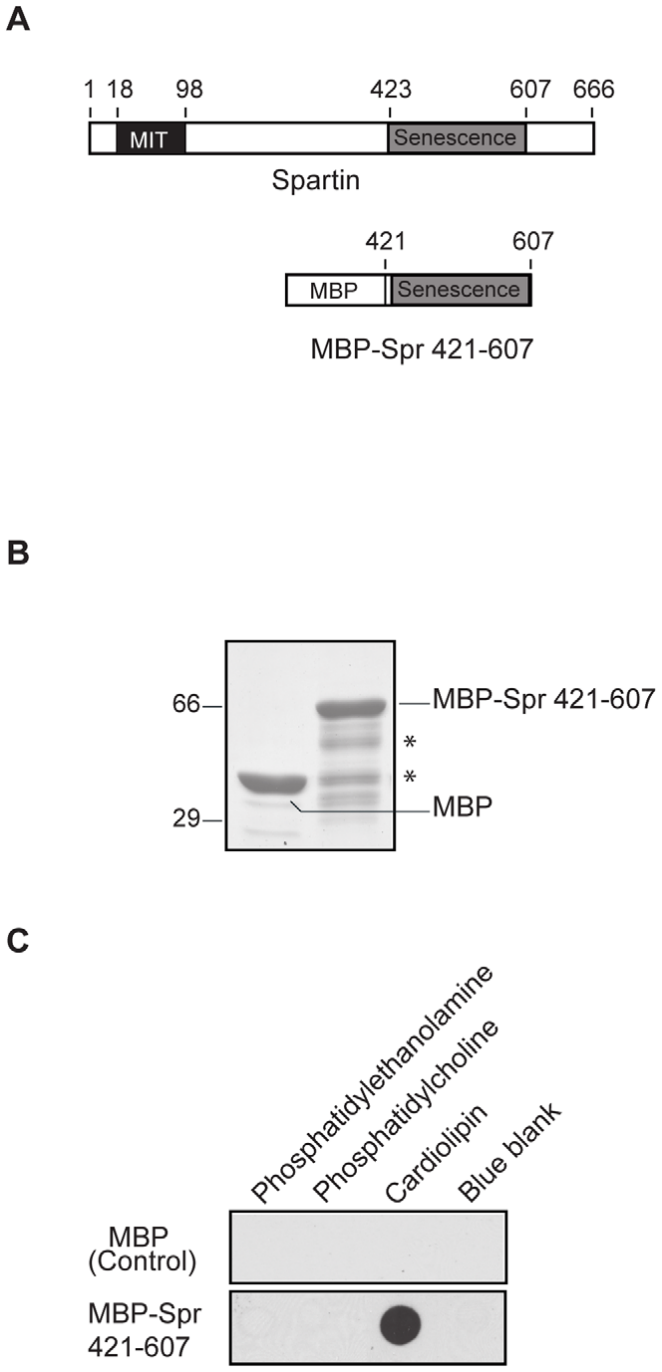
A plant-related senescence domain of spartin binds to cardiolipin. (**A**) Schematic diagrams of the full-length spartin with the MIT and the plant-related senescence domains and the MBP-spartin (421–607) construct encompassing the plant-related senescence domain. Numbers represent the amino acid residues, showing the boundaries of indicated domains. (**B**) Coomassie blue staining of affinity-purified MBP and MBP-spartin (421–607) separated on a polyacrylamide gel. The asterisk (*) indicates a degradation product of MBP-spartin (421–607). Sizes of protein standards are indicated to the left in kDa. (**C**) Detection of MBP-spartin (421–607) binding to cardiolipin but not to phosphatidylethanolamine or phosphatidylcholine in a lipid-protein overlay assay. MBP alone (negative control) did not associate with cardiolipin, phosphatidylethanolamine, or phosphatidylcholine.

Spartin protein has no mitochondrial targeting sequence and might associate with these organelles through the interaction of its C-terminus with proteins and/or phospholipids that reside in the mitochondria. The C-terminus of spartin encompasses the plant-related senescence domain that is conserved in many proteins in various species including *Arabidopsis thaliana* suggesting of its important function. We reasoned that this domain might bind to mitochondrial phospholipids. To test this hypothesis, we expressed and purified a maltose binding protein (MBP)-spartin (421–607) (which encompasses the entire plant-related senescence domain) fusion protein and MBP alone (Figure 2A and B). MBP-spartin (421–607) and MBP alone (used as a negative control) were applied in an *in vitro* protein-lipid overlay assay using nitrocellulose membranes with pre-spotted phospholipids. Using anti-MBP antibodies, we found that MBP-spartin (421–607) interacted with cardiolipin but not with two other major mitochondrial phospholipids, namely phosphatidylethanolamine and phosphatidylcholine (Figure 2C). The negative control (MBP alone) interacted with neither cardiolipin nor with phosphatidylethanolamine or phosphatidylcholine (Figure 2C). Overall, our results indicate that spartin interacts with mitochondria via its plant-related senescence domain, which binds to cardiolipin, a major phospholipid of the mitochondrial membrane.

### Spartin associates with outer mitochondrial membrane

**Figure 3:**
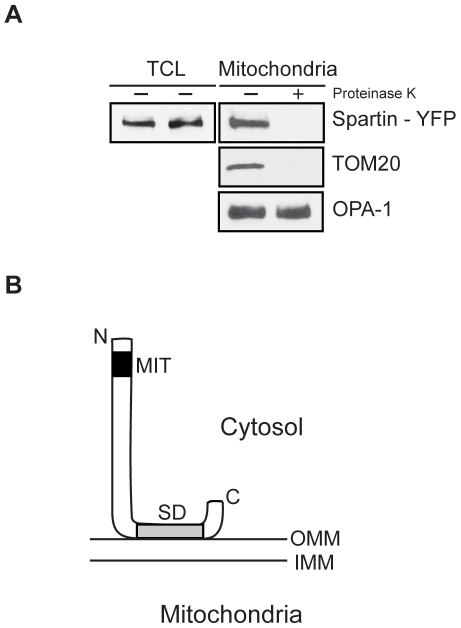
The topology of spartin's association with the mitochondrial membrane. (**A**) SK-N-SH cells were transfected with C-terminus tagged spartin-YFP, and spartin and mitochondrial fractions were incubated with or without proteinase K. The total cell lysates (TCL) were immunoblotted for YFP, and mitochondrial fractions were immunoblotted for YFP, TOM20 (in the outer mitochondrial membrane) and OPA-1 (in the inner mitochondrial membrane). (**B**) A proposed model of spartin's association with the outer mitochondrial membrane via its senescence domain (SD). OMM and IMM, the outer and inner mitochondrial membrane, respectively.

Cardiolipin is a major phospholipid in the inner mitochondria membrane, but it has been also found in the outer mitochondrial membrane [21]. To determine the topology of spartin in the mitochondria, we overexpressed spartin-YFP in SK-N-SH cells and isolated mitochondrial fractions. Those fractions were either treated or not treated with proteinase K followed by immunoblotting (Fig. 3A). Overexpressed spartin-YFP, TOM20, and OPA1 were all detected in the mitochondrial fraction not treated with proteinase K (Figure 3A). Enzymatic treatment eliminated detection of spartin-YFP and TOM20, which are anchored to the outer mitochondrial membrane. In contrast, OPA1, a resident protein of the intermembrane space, was still detected, indicating that the inner mitochondrial membrane was intact (Figure 3A). Overall, our results suggest that spartin is associated with the outer mitochondrial membrane (Figure 3B). Importantly, alpha-synuclein has also been reported to associate with cardiolipin and to locate to the outer mitochondria membrane [22].

### Depletion of spartin results in depolarization of the mitochondrial membrane

Our previous studies found that in fibroblasts derived from patients with Troyer syndrome there is a lack of expression of truncated spartin protein, implying that the pathology of this disease occurs via a loss-of-function mechanism [4]. Our present findings show that spartin associates with mitochondria through its binding to cardiolipin, a major mitochondrial phospholipid. These results prompted us to investigate mitochondrial function after knock down of spartin's expression. Specifically, we investigated the ΔΨ_m_ and ATP production in cells depleted of spartin. The mitochondrial membrane potential was monitored in live cells using a fluorescent probe, tetramethylrhodamine methyl ester (TMRM), as described in *Materials and Methods*.

**Figure 4:**
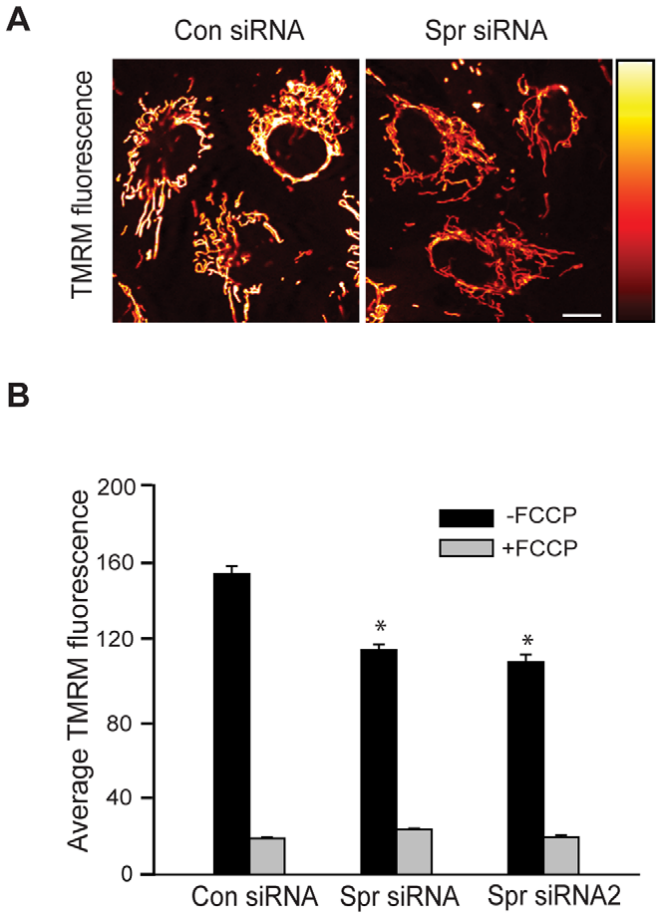
Spartin depletion causes depolarization of mitochondrial membrane potential. (**A**) Representative images showing TMRM fluorescence in SK-N-SH cells treated with control (left panel) or spartin siRNA (right panel). The pseudocolor bar represents an intensity scale, with black as minimum and bright yellow as maximum intensity. (**B**) The graph shows the average pixel fluorescence intensity of TMRM in cells treated with control or spartin siRNA. Cells were either treated or not treated with FCCP as indicated. The data represent mean ± S.E.M in 150 cells for each condition from three independent experiments (*p>0.01).

SK-N-SH cells treated with control or spartin siRNA were analyzed by an observer who was ‘blind’ to the experimental conditions. We found that the mitochondrial membrane was depolarized in cells treated with spartin siRNA1 compared with control siRNA-treated cells (Figure 4A). Quantitative analysis from three independent experiments revealed that depletion of spartin with siRNA1 resulted in ∼25% lower average pixel fluorescence intensity of TMRM compared with cells expressing physiological levels of spartin (154±3.9 *vs*. 114±2.9, control *vs*. spartin siRNA1-treated cells, respectively, p<0.01; Figure 4B). Similar results were found when cells were treated with spartin siRNA2 (154±3.9 *vs*. 108±3.9, control *vs*. spartin siRNA2-treated cells, respectively, p<0.01; Figure 4B). Importantly, all cells treated with carbonyl cyanide 4-(trifluoromethoxy)-phenylhydrazone (FCCP), a mitochondrial uncoupler, showed very low fluorescence intensity of TMRM, reflecting a collapsed **Δ**Ψ_m_ (Figure 4B). We also examined the mitochondrial membrane potential in primary cortical neurons isolated from the wild type (WT) and *Spg20* knockout mice. The lack of spartin's expression in these mutant mice was confirmed by immunoblotting homogenate tissue from the brain, heart, and liver of these mice (Figure S2; Text S1; data not shown). We determined that the mitochondria membrane in cortical neurons from mutant mice was depolarized compared to WT cortical neurons. Average TMRM fluorescence intensity in cortical neurons from *Spg20* KO mice was ∼24% lower than that of neurons derived from WT mice (85.85±1.82 *vs*. 65.73±1.68, WT *vs*. mutant neurons, p<0.01) (Figure S5A; Text S1).

We also examined whether spartin depletion alters the levels of ATP production. We found that spartin siRNA-treated SK-N-SH cells showed lower levels of ATP production compared with control siRNA-treated cells (Figure S4). However, these changes were not statistically significant. Overall, our results suggest that spartin plays a role in maintaining the mitochondrial membrane potential.

### High intracellular calcium levels reduce the mitochondrial uptake of calcium and depolarize mitochondrial membrane in spartin-depleted cells

**Figure 5:**
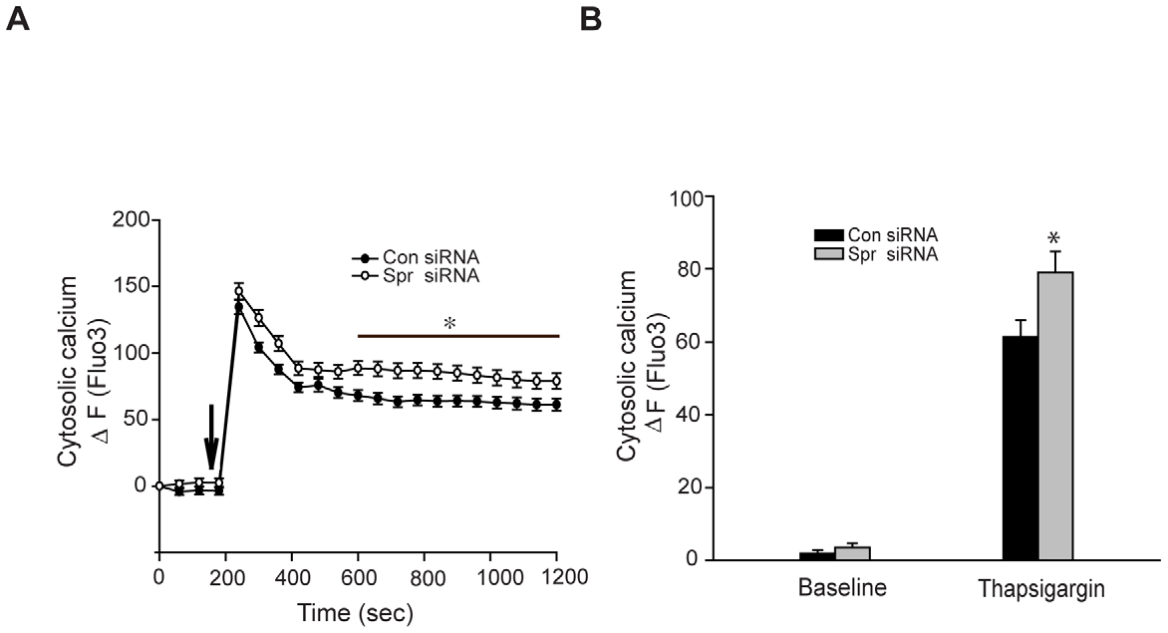
Thapsigargin treatment induced high levels of intracellular calcium measured by Fluo3-AM. (**A**) Fluorescence changes of Fluo3 are shown as ΔF and reflect the cytosolic Ca^2+^ levels. Spartin (white circles) and control (black circles) siRNA-treated cells were incubated with Fluo3-AM and stimulated (indicated by the arrow) with 1 µM of thapsigargin. An asterisk (*) indicates a significant difference in cytosolic Ca^2+^ levels between spartin and control siRNA-treated cells at the indicated time points. (**B**) The graph shows quantification of relative fluorescence changes of Fluo3 signifying cytosolic Ca^2+^ levels. Analysis was performed in cells treated with control (black bars) or spartin (gray bars) siRNA at baseline (before stimulation with thapsigargin) or at 600 sec after the collection of the first image. The data represent mean ± S.E.M in 70 cells from two independent experiments (*p>0.05).

**Figure 6:**
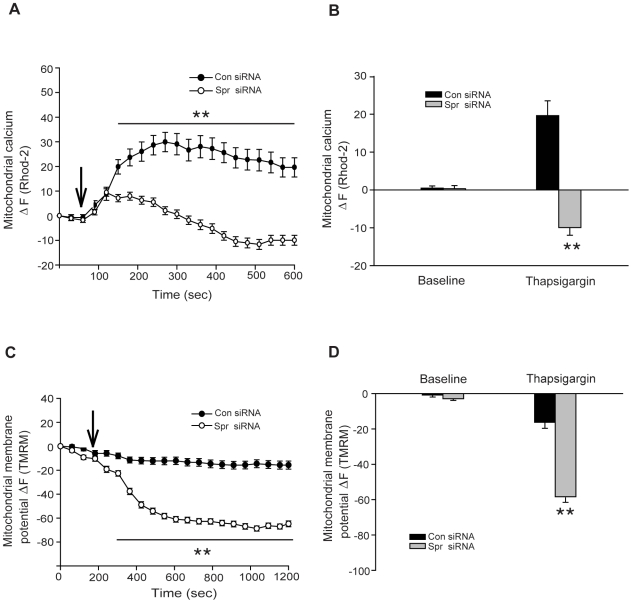
Increased intracellular Ca^2+^ levels result in mitochondrial dysfunction in SK-N-SH cells depleted of spartin. (**A**) Fluorescence changes of Rhod-2 intensities are shown as ΔF and signify the mitochondrial Ca^2+^ levels. Control (black circles) and spartin (white circles) siRNA-treated cells were incubated with Rhod-2-AM and stimulated (indicated by the arrow) with 1 µM of thapsigargin. Error bars represent S.E.M. (**p<0.001). (**B**) The graph shows quantification of relative fluorescence changes of Rhod-2 signifying mitochondrial Ca^2+^ levels. Analysis was performed in cells treated with control (black bars) or spartin (gray bars) siRNA at baseline (before stimulation with thapsigargin) or at 600 sec after the start of the experiment. **p<0.001. The data represent mean± S.E.M in 80 cells from three different experiments. (**C**) Fluorescence changes of TMRM intensities are shown as ΔF and determine the mitochondrial membrane potential. Black and white circles as in (A). Cells were stimulated (indicated by the arrow) with 1 µM of thapsigargin. **p<0.001. (**D**) The graph shows quantification of relative fluorescence changes of TMRM signifying the levels of mitochondrial membrane potential. Analysis was done in cells treated with control (black bars) or spartin (gray bars) siRNA at baseline (before stimulation with thapsigargin) and at 600 sec after taking the first image. The data represent mean ± S.E.M in 100 cells from four independent experiments (**p<0.001).

Mitochondria and the endoplasmic reticulum (ER) play a critical role in intracellular Ca^2+^ homeostasis (reviewed in [23]). Evidence indicates that the loss of mitochondrial Ca^2+^ buffering capacity might be an important factor in the pathophysiology of Huntington's disease, a neurodegenerative disorder that affects neurons in the striatum [14], [24]. Thus, to determine whether spartin has a role in the homeostasis of mitochondrial Ca^2+^, we induced high intracellular Ca^2+^ levels by treating cells with thapsigargin. Thapsigargin increases intracellular Ca^2+^ levels by depleting the ER Ca^2+^ stores and restraining the flux of Ca^2+^ to the ER by inhibiting sarcoplasmic/endoplasmic reticular Ca^2+^ ATPase [25]. Thapsigargin treatment produces experimental conditions by which it is possible to selectively investigate mitochondrial Ca^2+^ uptake without interference from the ER. We measured the intracellular and mitochondrial Ca^2+^ levels using fluorescent probes, Fluo3- AM and Rhod-2-AM, respectively, in cells treated with thapsigargin (1 µM). The experiment was conducted for 1200 seconds, and from 600 to 1200 sec, we found significantly higher levels of Fluo3 intensity in spartin siRNA1-treated cells than in control siRNA-treated cells (Figure 5A). At the 600-sec time point spartin-depleted cells and control cells showed average Fluo3 fluorescence pixel intensity of 79.9±5.8 and 61.2±4.5 (p<0.05; Figure 5B) average fluorescence pixel intensity of Fluo3, respectively. These results indicate that spartin depletion results in high levels of intracellular Ca^2+^. In contrast, the Ca^2+^ levels in mitochondria determined by using Rhod-2 were significantly lower in spartin-depleted cells compared with control cells after stimulation with thapsigargin (Figure 6A). At 600 sec, we detected about 30% lower Rhod-2 intensity in spartin siRNA1-treated cells compared with cells expressing physiological levels of spartin (−9.9±2.0 *vs*. 19.6±3.9 normalized Rhod-2 ratios, p<0.001; Figure 6B). Similar results were obtained in cells treated with spartin siRNA2 (Figure S5A and S5B). In these experiments, we acquired images for a total duration of 600 sec because, in preliminary studies, we observed bleaching of Rhod-2 fluorescence intensity after 600 sec of taking images. Thus, in order not to have confounding results, we restricted the imaging time to 600 sec.

Mitochondrial Ca^2+^ uptake during thapsigargin exposure was ensured using Ruthenium red, a specific inhibitor of mitochondrial uniporter [26]. Cells were incubated with 10 µM of Ruthenium red for 1 hr, and Rhod-2 fluorescence was measured before and after thapsigargin treatment (Text S1). We observed a significant decrease in Rhod-2 fluorescence intensity in the presence of Ruthenium red in both control and spartin siRNA-treated cells compared with thapsigargin alone (Figure S5A). This strongly suggests that the Ca^2+^ uptake in both treatment groups occurs via the mitochondrial uniporter.

Because lower calcium levels in mitochondria are correlated with depolarization of the mitochondrial membrane [27], we measured **Δ**Ψ_m_ in cells depleted of spartin after thapsigargin (1 µM) treatment by using TMRM. As shown in Fig. 6C, thapsigargin treatment led to higher depolarization of mitochondrial membrane in cells depleted of spartin compared with cells expressing endogenous spartin. Data from four independent experiments revealed that cells with knocked-down spartin had four-fold lower TMRM fluorescence intensity than cells expressing physiological levels of spartin (Figure 6D). Specifically, quantitative analysis at 600 sec showed that TMRM average fluorescence intensity was −15.2±3.3 and −60±2.0 (normalized TMRM ratios, p<0.001; Figure 6D) in control siRNA and spartin siRNA1-treated cells, respectively. The TMRM average fluorescence intensity was also significantly lower when cells were treated with spartin siRNA2 compared to cells treated with control siRNA (Figure S5C and D).

To confirm these findings in another cellular model (i.e., lack of spartin's expression) of Troyer syndrome, we used neurons derived from *Spg20* KO mice. Thapsigargin treatment resulted in a higher depolarization of mitochondrial membrane in primary cortical neurons derived from *Spg20* KO mice compared to neurons obtained from WT mice. The quantitative analysis revealed 1.8-fold lower TMRM fluorescence intensity in mutant neurons than in WT neurons. At 1200 sec, the average TMRM fluorescence intensity was −30.9±2.6 and −56.8±3.5 (p<0.001; Figure S3C) in neurons derived from the WT and *Spg20* KO mice, respectively. Together, our data indicate that spartin plays an important role in maintaining mitochondrial Ca^2+^ buffering capacity and mitochondrial membrane potential.

## Discussion

In this study, we identified the role of the Troyer syndrome protein spartin in mitochondrial functions. We demonstrated the binding of the conserved plant-related senescence domain of spartin to cardiolipin, a mitochondrial phospholipid; this might be a major means by which spartin associates with the mitochondria. Human neuroblastoma cells depleted of spartin and cortical neurons obtained from *Spg20* KO mice showed depolarized mitochondrial membrane. In addition, knockdown of spartin reduced mitochondrial Ca^2+^ influx upon thapsigargin stimulation. Our findings suggest that spartin is an important player in the physiological function of mitochondria and that lack of spartin's expression in patients with Troyer syndrome might cause impaired mitochondrial calcium handling, which could contribute to the pathophysiology of the disease.

The subcellular localization of spartin in mitochondria has been controversial. Two studies by Byrne's laboratory have shown, by immunofluorescence and immunoblotting, that overexpressed and endogenous spartin is present in the mitochondria [6], [11]. However, a study by Eastman and colleagues did not replicate those findings [12]. By using both immunofluorescence and differential centrifugation of cells expressing endogenous spartin, we found that spartin localized to cytoplasm and mitochondria. Furthermore, using a biochemical assay we showed that the C-terminus of spartin is responsible for its association with mitochondria. These findings agree with an earlier immunofluorescence study demonstrating that the C-terminus of overexpressed spartin colocalizes with mitochondria [6].

Proteins that are not permanently located in the mitochondria due to the lack of a mitochondrial targeting signal associate with the mitochondria by two major, non-mutually exclusive mechanisms: 1) they bind to the proteins that reside in the mitochondria [28] and/or 2) they bind to phospholipids enriched in the mitochondrial membranes [22], [29]. We found that spartin, through its plant-related senescence domain, binds to cardiolipin, a phospholipid that is present mostly in the inner [30], and to a lesser degree in the outer mitochondrial membrane [21]. Our experiments examining the topology of spartin revealed that it associates with the outer mitochondria membrane. This finding together with the presence of a large pool of spartin in the cytoplasm [7], [8], suggests that spartin might transiently bind to cardiolipin and/or protein(s) located on the outer mitochondrial membrane. This would also be in agreement with our previous findings showing that spartin is a highly mobile protein [7].

Spartin is a multifunctional protein [11] and localizes to many subcellular compartments [6]–[9]. The localization of spartin to some specific organelles is transient and becomes evident after cells are treated with compounds that facilitate a particular physiological change. For example, treatment of cells with epidermal growth factor, which induces synchronized endocytosis in serum-starved cells, facilitates distribution of spartin to the endosomes [7], [8]. When cells are treated with oleic acid, it causes the formation of lipid droplets and causes spartin to relocate from the cytoplasm to those lipid droplets [8], [12]. In cells grown in regular medium with serum, endogenous spartin has been reported to localize to *trans*-Golgi [9], midbodies during cell division [9], [31], and to mitochondria (present studies). Recently, it was reported that spartin affects lipid droplet turnover [12], [13], and in the present study we show that spartin also regulates calcium uptake to mitochondria. Thus, spartin's localization to the lipid droplets and mitochondria has biological functions and further supports the multifaceted role of spartin in the biology of the cell.

In addition to spartin, other proteins were originally found to be mostly present in the cytoplasm and then were also located to lipid droplets as well as mitochondria. For example, similar to spartin, wild-type and mutant A53T alpha-synuclein (which causes a dominant- negative form of Parkinson's disease), and protein kinase C (PKC) δ re-distribute from the cytoplasm to the lipid droplets after cells are treated with oleic acid [32], [33]. Whereas PKC δ translocates from the cytoplasm to the mitochondria after cells are treated with 12-*O*-tetradecanoylphorbol-13-acetate [34], a pool of alpha-synculein associates with mitochondria at steady state [35], [36]. Similar to spartin, alpha-synuclein binds to cardiolipin and is associated with the outer mitochondrial membrane [22].

It has been reported that the C-terminus of spartin is responsible for its association with lipid droplets in the presence of oleic acid [12] and with the mitochondria at steady state [6]. Spartin associates with lipid droplets via binding to the TIP-47 protein [12], a structural protein in lipid droplets [37]. However, the amino acid region that is responsible for spartin's binding to the TIP-47 protein remains to be determined. In the present study, we determined that spartin through its plant-related senescence domain (amino acids 423–607) binds to cardiolipin, suggesting that this might be the means by which spartin associates with the mitochondria.

An important finding of our study is that acute depletion of spartin by siRNA significantly decreased mitochondrial Ca^2+^ uptake capacity. It is well known that mitochondrial Ca^2+^ buffering is controlled mainly by mitochondrial membrane potential, which provides the driving force for Ca^2+^ to enter the matrix via the Ca^2+^ uniporter (reviewed in [38]). In pathological conditions, the efflux of Ca^2+^ from the mitochondrion occurs via the mitochondrial permeability transition pore (MPTP), which is opened by several factors, including depolarized mitochondrial membrane potential, high levels of ROS in mitochondria, and/or high levels of Ca^2+^ in the matrix [39]. Thus, two possible scenarios could account for poor mitochondrial Ca^2+^ handling in cells depleted of spartin. First, the lack of spartin's expression might lead to diminished Ca^2+^ uptake through the uniporter because of the depolarized mitochondrial membrane potential. Alternatively, a rapid influx of calcium might open the MPTP thereby permitting the release of Ca^2+^ to the cytosol. The latter mechanism has been reported to be responsible for the low uptake of Ca^2+^ by mitochondria in striatal neurons derived from a murine model of Huntington's disease [40]. Treatment with MPTP inhibitors significantly improved mitochondrial Ca^2+^ uptake in mutant huntingtin-expressing striatal neurons [40]. We are currently investigating molecular mechanisms by which spartin might regulate Ca^2+^ uptake to the mitochondria.

In summary, we demonstrate the localization of spartin to mitochondria and the functional role of spartin in maintaining mitochondrial Ca^2+^ buffering capacity and mitochondrial membrane potential. Spartin is also involved in trafficking of cargo receptors [7], [8] and in the turnover of lipid droplets [12], [13]. It is reasonable to suggest that several cellular dysfunctions due to lack of spartin's expression contribute to a complex constellation of phenotypic symptoms present in patients with Troyer syndrome. We have just begun to determine specific functions of spartin at the cellular level, and the future experiments will parse out the different aspects of spartin's biological role, including its role in mitochondrial function, on the integrity of the corticospinal axons.

## Materials and Methods

### Cell Culture and Transfection

SK-N-SH cells were maintained in Minimal Essential Medium (MEM) (Mediatech, Inc., Manassas, VA) supplemented with 10% fetal bovine serum (Gemini BioProducts, West Sacramento, CA) and essential amino acids (Invitrogen, Carlsbad, CA). The transfections with DNA plasmids and siRNA were performed using Lipofectamine and Lipofectamine RNAiMAX (Invitrogen), respectively, according to the manufacturer's instructions. The hemagglutinin (HA)-tagged full-length spartin, HA-spartin 1–408, and spartin-yellow fluorescence protein (YFP) in YFP-N1 were described previously [7]. All functional experiments were performed using control and spartin siRNA1 and siRNA2, the sequences of which we described previously [7]. To generate the pGW1-HA-tagged human spartin 409–666 construct, we used PCR, and the amplified DNA was cloned in-frame to an *EcoR*I site into the pGW1-HA vector.

### Antibodies and Reagents

To generate polyclonal antibodies against human spartin, we subcloned human spartin (108–367) into a pGEX-6p-1 vector and expressed the glutathione S-transferase (GST) fusion protein in BL21 *E. coli* bacteria as described previously [10]. The protein was purified, digested with PreScission Protease (GE Healthcare, Waukesha, WI), eluted, and injected into guinea pigs to produce polyclonal antisera (Veritas Labs, Rockville, MD). We purified the anti-spartin IgG fraction using protein A-Sepharose (Sigma-Aldrich, St. Louis, MO). The following primary antibodies were used: mouse monoclonal anti-optic atrophy 1 (OPA1; BD Biosciences, San Jose, CA), mouse anti-early endosome antigen 1 (EEA1; BD Biosciences), anti-phospholipase C-γ (PLC- γ; Upstate Biotechnology, Lake Placid, NY), rabbit polyclonal anti-HA-epitope (Abcam, Cambridge, MA), mouse monoclonal anti-translocase of the outer membrane 20 (TOM20; BD Biosciences), mouse monoclonal anti-β-tubulin (clone 6G7) (Developmental Studies Hybridoma Bank, University of Iowa, IA), mouse anti-YFP (Covance, Princeton, NJ) and mouse anti-maltose binding protein (MBP) (New England BioLabs, Ipswich, MA). The anti-rabbit, anti-mouse, or anti-guinea pig antibodies conjugated to horseradish peroxidase (HRP) were from Thermo Fischer Scientific (Waltham, MA). The secondary antibodies used for immunofluorescence included anti-mouse, anti-rabbit, or anti-guinea pig conjugated to Alexa Fluor 488 or 555 (Invitrogen). Thapsigargin was purchased from Enzo Life Sciences (Plymouth Meeting, PA) and FCCP and proteinase K were obtained from Sigma-Aldrich.

### Subcellular Fractionation and Protease Digest

To detect endogenous spartin in different subcellular fractions, cells were washed with ice-cold phosphate-buffered saline (PBS; pH 7.4) containing 1 mM EDTA, harvested, and resuspended in buffer A (210 mM mannitol, 70 mM sucrose, 1 mM EDTA, 10 mM HEPES, pH 7.5 with protease inhibitors) then disrupted using a Dounce homogenizer (30 strokes). The homogenate was centrifuged at 900× *g* for 5 min, and the supernatant was recentrifuged at 900× *g* for 5 min to remove the unbroken cells and nuclei. The post-nuclear fraction was centrifuged at 10,000× *g* for 10 min, yielding a heavy-membrane fraction (HM) and supernatant that was subsequently centrifuged at 100,000× *g*, generating light-membrane (LM) and cytosolic fractions. The protein concentrations were measured by bicinchoninic acid protein assay (Thermo Fisher Scientific), and 30 µg of total protein from each fraction was resolved on 8% SDS-PAGE gel. The presence of spartin, OPA1 (a mitochondrial marker), and phospholipase C-γ (a cytosolic marker) was analyzed by immunoblotting. To determine the localization of spartin in the mitochondrial membranes, SK-N-SH cells were overexpressed with spartin-YFP vector. The isolated mitochondrial fractions were either treated or not treated with proteinase K (50 µg/ml) on ice for 30 min, and the reaction was stopped by adding 5 mM of phenylmethylsulfonyl fluoride (PMSF) as described previously [41]. Equal amount of proteins (30 µg) from the mitochondrial fraction treated with or without the enzyme were resolved on SDS-PAGE gradient gel (Invitrogen). The presence of overexpressed spartin-YFP, OPA1 (localized in the mitochondrial intermembrane space), and TOM20 (localized in the outer mitochondrial membrane) was analyzed by immunoblotting.

### Lipid-Protein Overlay Assay

Spartin construct 421–607 was cloned into a pMal-5 vector, using *Bam*HI and *Eco*RI restriction sites at the 5′ and 3′- ends, respectively. The MBP–spartin 421–607 or MBP alone was expressed in *E. coli* BL21 cells, incubated with amylase resin (New England Bio*Labs*) overnight at 4°C while rocking, and then eluted according to the manufacturer's instructions. The nitrocellulose membrane with spotted lipids, including cardiolipin and a solvent blank control, were purchased from Echelon Biosciences (Salt Lake City, UT). The membrane was wetted in molecular biology grade water, then equilibrated with Tris-buffered saline Tween-20 (TBST) for 5 min and subsequently incubated with blocking solution as described previously [42]. Then the membrane was incubated with 2 µg/ml of MBP-spartin 421–607 or MBP in TBST overnight at 4°C. The following day the membrane was washed in TBST, incubated with primary anti-MBP antibody for 1 hr, washed, and incubated with anti-mouse antibody conjugated with HRP. After extensive washes, the membrane was incubated with enhanced chemiluminescence (Thermo Fisher Scientific) and exposed to film.

### Immunofluorescence

SK-N-SH cells were grown on glass cover slips, fixed with 4% paraformaldehyde for 25 min, and processed as described previously [10]. Cover slips were mounted with ProLong Antifade reagent (Invitrogen), and images were acquired using a Zeiss LSM-510 confocal microscope with a 63× 1.4 NA Plan Apochromat oil immersion objective at 1024×1024 resolution. The images were processed with Adobe Photoshop 7.0 software (Adobe, San Jose, CA).

### Measurement of Mitochondrial Membrane Potential in Live Cells

Mitochondrial membrane potential was measured by the potentiometric fluorescent probe, tetramethylrhodamine methyl ester (TMRM; Invitrogen), which accumulates in the mitochondrial inner and outer membrane based on ΔΨ_m_ and can be detected using live cell imaging [43]–[45]. Cells treated with control or spartin siRNA for 48 hrs were incubated with TMRM (50 nM) in Tyrode's buffer (TB) (145 mM NaCl, 5 mM KCl, 10 mM glucose, 1.5 mM CaCl_2_, 1 mM MgCl_2_, 10 mM HEPES, pH 7.4) for 30 min at room temperature. The same concentration of TMRM was present throughout the experiment. The culture dishes with a glass bottom (MatTek Corporation, Ashland, MA) were placed over the mounting chamber, and a field containing 15–20 cells was selected. Fluorescence imaging was performed using confocal microscopy (LSM 510, Carl Zeiss MicroImaging Inc, Thornwood, NY). The observer who acquired the images was ‘blind’ to the experimental conditions. Images from randomly selected fields were collected by using a 40× water immersion objective at 514/570 nm excitation/emission with an argon laser at 5% transmission and 256×256 resolution. Images were acquired with identical instrument settings across all samples to ensure the comparability between experimental groups. The fluorescence images were collected for 1 sec at an interval of 59 sec and axial resolution of 3.0 µm, to increase the optical thickness, and a pixel depth of 12 bits. About 25–30 mitochondrial structures were chosen as regions of interest (ROIs) in each cell and pixel intensity of TMRM fluorescence in these regions was averaged after background subtraction. The decrease in average pixel intensity of TMRM fluorescence in mitochondrial regions of interest (ROIs) was interpreted to signify the depolarization of the mitochondrial membrane potential. Mitochondrial localization of TMRM was confirmed by using a protonophore FCCP, which eliminates the TMRM fluorescence from mitochondria by collapsing the mitochondrial membrane potential. In our time series experiment, after subtracting the background fluorescence, the changes in TMRM fluorescence intensity were calculated using the formula ΔF = F−F_0_/F_0_×100, where F_0_ is the initial fluorescence and F is the fluorescence intensity at any time point [46], [47].

### Intracellular and mitochondrial Ca^2+^ measurements

Intracellular and mitochondrial Ca^2+^ were measured with the Ca^2+^-sensitive fluorescent probes Fluo3- acetoxymethyl (AM) ester and Rhod-2 AM (Invitrogen) , respectively, as described previously with minor modifications [48]–[50]. Briefly, SK-N-SH cells grown in dishes with a glass bottom (MatTek Corporation) were washed 3 times with TB and incubated with Fluo-3AM (5 µM) containing 0.02% pluronic acid in TB at 37°C for 30 min [50], [51]. Then cells were washed 4 times with TB and incubated in the dark for 45 min to complete de-esterification of AM ester by intracellular esterases to ensure the binding of the probe to free intracellular Ca^2+^ [52]. To measure the mitochondrial Ca^2+^, Rhod-2 AM was reduced to dihydrorhod-2 by adding a small amount of sodium borohydrite (NaBH_4_, a stock of 1 mg/ml was prepared in methanol) which is known to increase the mitochondrial loading of the probe [49], [53]. Cells were incubated with 5 µM of reduced Rhod-2 AM containing 0.02% pluronic acid for 60 min in TB then washed 4 times with TB and incubated in MEM for another 6 hrs. We determined the 6-hr incubation time for SK-N-SH cells empirically, because during that time the Rhod-2 dye was present predominantly in the mitochondria, as indicated by its colocalization with MitoTracker Green FM (100 nM). A field containing a minimum of 15–20 cells was selected for the experiments. The images were acquired using 488/515 nm and 561/580 nm, excitation/emission for Fluo-3 and Rhod-2, respectively, with the laser power and resolution as described for mitochondrial membrane potential. The fluorescence images were collected for 1 sec at an interval of 59 sec or 29 sec to measure the cytosolic and mitochondrial Ca^2+^ levels at an axial resolution of 3 µm and a pixel depth of 12 bits. The change in fluorescence intensity (ΔF) was calculated similarly to that for TMRM.

### Statistical analysis

Data analysis was carried out using Sigma plot software (Systat Software Inc, Chicago, IL). The data were represented as mean ± SEM, calculated from 3–5 experiments. To calculate statistical significance, we used Student's *t*-test, and *p*≤0.05 was considered significant.

### Detection of ATP

ATP levels were measured using an ATPLite kit (Perkin Elmer) according to the manufacture's instructions. The kit contains luciferin and luciferase reagents to detect ATP by bioluminescence. Luminescence was measured in a Bio-Tek luminometer. [54]

## Supporting Information

Figure S1 **The association of spartin with mitochondria using fractionation.** (**A**) Schematic diagrams of HA-tagged full-length spartin and deletion constructs encompassing microtubule interacting and trafficking (MIT) and/or plant-related senescence domain studied in a mitochondrial fraction. Numbers represent the amino acid residues, showing the boundaries of MIT and plant-related senescence domain. (**B**) SK-N-SH cells were transfected with indicated constructs of spartin, and the post-nuclear total homogenates (T) were fractionated into soluble (S) and mitochondria-enriched heavy-membrane fractions (HM) and immunoblotted with anti-HA antibodies. Sizes of protein standards are indicated to the left in kDa.
(TIF)

Figure S2 **Analysis of expression of spartin in brain tissue from WT and** ***Spg20*** **KO mice.** Brain tissues from wild type (WT) and *Spg20* KO mice were homogenized and immunoblotted with anti-spartin (upper panel) and β- tubulin antibodies (lower panel).
(TIF)

Figure S3 **Depolarization of mitochondrial membrane potential in cultured primary cortical neurons derived from** ***Spg20*** **KO mice.** (**A**) Average pixel fluorescence intensity of TMRM from randomly selected mitochondrial regions in WT and *Spg20* KO primary cortical neurons. Neurons were treated or not treated with the mitochondrial uncoupler, FCCP. (B) Changes in TMRM fluorescence intensity before and after treatment with 1 µM thapsigargin (indicated by arrow) in WT (black circle) and *Spg20* mutant (white circle) neurons. (C) Bar graphs showing the relative fluorescence changes of TMRM representing the levels of mitochondrial membrane potential. Analysis was carried out in WT (black bars) or *Spg20* mutant (gray bars) neurons at baseline (before stimulation with thapsigargin) and at 1200 sec after taking the first image. The data represent mean ± S.E.M in 75 neurons from three independent experiments (**p<0.001).
(TIF)

Figure S4 **The levels of ATP in SK-N-SH cells treated with control or spartin siRNA.** Cells were treated with siRNA for 48 hrs and ATP levels were measured using ATPlite luminescence assay kit (PerkinElmer) according to the manufacturer's protocol. Data represent mean ± SEM luminescence in triplicate treatment groups.
(TIF)

Figure S5 **High intracellular Ca^2+^ levels cause mitochondrial dysfunction in spartin depleted SK-N-SH cells.** (**A**) Changes in Rhod-2 fluorescence intensities (ΔF) upon 1 µM thapsigargin exposure in control siRNA (black circle) and spartin (white circle) siRNA2-treated cells. Fluorescence changes of Rhod-2 intensities were also measured in siRNA treated cells in the presence of mitochondrial Ca^2+^ uniporter blocker, Ruthenium red, prior to their stimulation with thapsigargin. Control siRNA (black triangles) and spartin siRNA (white triangles) depict changes in Rhod-2 fluorescence intensity upon thapsigargin exposure in cells treated with Ruthenium red. (**B**) The bar graph shows quantification of relative changes in Rhod-2 fluorescence intensity indicating the mitochondrial Ca^2+^ levels. Analysis was performed at baseline (before thapsigargin treatment), at 600 sec after the start of the experiment in control (black bars) and spartin siRNA2 (grey bars)-treated cells. Treatment groups are indicated on the X-axis. The data represent mean± S.E.M in 80 cells from two different experiments (**p<0.001). (**C**) Changes in TMRM fluorescence intensity (ΔF) upon 1 µM thapsigargin treatment (indicated by the arrow) in control (black circles) and spartin siRNA2 (white circles)-treated cells. (**D**) Bar graph representing the quantification of relative fluorescence changes of TMRM (ΔF) at baseline (before thapsigargin treatment) and at 600 sec after taking the first image in control (black bars) and spartin siRNA2 (grey bars) treated cells. The data represent mean ±S.E.M in 100 cells from three independent experiments (**p<0.001).
(TIF)

Text S1 (DOC)

## References

[1] Crosby AH, Proukakis C (2002). Is the transportation highway the right road for hereditary spastic paraplegia?. Am J Hum Genet.

[2] Cross HE, McKusick VA (1967). The Troyer syndrome. A recessive form of spastic paraplegia with distal muscle wasting.. Arch Neurol.

[3] Patel H, Cross H, Proukakis C, Hershberger R, Bork P (2002). SPG20 is mutated in Troyer syndrome, an hereditary spastic paraplegia.. Nat Genet.

[4] Bakowska JC, Wang H, Xin B, Sumner CJ, Blackstone C (2008). Lack of spartin protein in Troyer syndrome: a loss-of-function disease mechanism?. Arch Neurol.

[5] Ciccarelli FD, Proukakis C, Patel H, Cross H, Azam S (2003). The identification of a conserved domain in both spartin and spastin, mutated in hereditary spastic paraplegia.. Genomics.

[6] Lu J, Rashid F, Byrne PC (2006). The hereditary spastic paraplegia protein spartin localises to mitochondria.. J Neurochem.

[7] Bakowska JC, Jupille H, Fatheddin P, Puertollano R, Blackstone C (2007). Troyer syndrome protein spartin is mono-ubiquitinated and functions in EGF receptor trafficking.. Mol Biol Cell.

[8] Edwards TL, Clowes VE, Tsang HT, Connell JW, Sanderson CM (2009). Endogenous spartin (SPG20) is recruited to endosomes and lipid droplets and interacts with the ubiquitin E3 ligases AIP4 and AIP5.. Biochem J.

[9] Robay D, Patel H, Simpson MA, Brown NA, Crosby AH (2006). Endogenous spartin, mutated in hereditary spastic paraplegia, has a complex subcellular localization suggesting diverse roles in neurons.. Exp Cell Res.

[10] Bakowska JC, Jenkins R, Pendleton J, Blackstone C (2005). The Troyer syndrome (SPG20) protein spartin interacts with Eps15.. Biochem Biophys Res Commun.

[11] Milewska M, McRedmond J, Byrne PC (2009). Identification of novel spartin-interactors shows spartin is a multifunctional protein.. J Neurochem.

[12] Eastman SW, Yassaee M, Bieniasz PD (2009). A role for ubiquitin ligases and Spartin/SPG20 in lipid droplet turnover.. J Cell Biol.

[13] Hooper C, Puttamadappa SS, Loring Z, Shekhtman A, Bakowska JC (2010). Spartin activates atrophin-1-interacting protein 4 (AIP4) E3 ubiquitin ligase and promotes ubiquitination of adipophilin on lipid droplets.. BMC Biol.

[14] Panov AV, Gutekunst CA, Leavitt BR, Hayden MR, Burke JR (2002). Early mitochondrial calcium defects in Huntington's disease are a direct effect of polyglutamines.. Nat Neurosci.

[15] Manfredi G, Xu Z (2005). Mitochondrial dysfunction and its role in motor neuron degeneration in ALS.. Mitochondrion.

[16] Casari G, Rugarli E (2001). Molecular basis of inherited spastic paraplegias.. Curr Opin Genet Dev.

[17] Hansen JJ, Durr A, Cournu-Rebeix I, Georgopoulos C, Ang D (2002). Hereditary spastic paraplegia SPG13 is associated with a mutation in the gene encoding the mitochondrial chaperonin Hsp60.. Am J Hum Genet.

[18] Casari G, De Fusco M, Ciarmatori S, Zeviani M, Mora M (1998). Spastic paraplegia and OXPHOS impairment caused by mutations in paraplegin, a nuclear-encoded mitochondrial metalloprotease.. Cell.

[19] Atorino L, Silvestri L, Koppen M, Cassina L, Ballabio A (2003). Loss of m-AAA protease in mitochondria causes complex I deficiency and increased sensitivity to oxidative stress in hereditary spastic paraplegia.. J Cell Biol.

[20] Cabiscol E, Belli G, Tamarit J, Echave P, Herrero E (2002). Mitochondrial Hsp60, resistance to oxidative stress, and the labile iron pool are closely connected in Saccharomyces cerevisiae.. J Biol Chem.

[21] de Kroon AI, Dolis D, Mayer A, Lill R, de Kruijff B (1997). Phospholipid composition of highly purified mitochondrial outer membranes of rat liver and Neurospora crassa. Is cardiolipin present in the mitochondrial outer membrane?. Biochim Biophys Acta.

[22] Cole NB, Dieuliis D, Leo P, Mitchell DC, Nussbaum RL (2008). Mitochondrial translocation of alpha-synuclein is promoted by intracellular acidification.. Exp Cell Res.

[23] Szabadkai G, Duchen MR (2008). Mitochondria: the hub of cellular Ca2+ signaling.. Physiology (Bethesda).

[24] Gizatullina ZZ, Lindenberg KS, Harjes P, Chen Y, Kosinski CM (2006). Low stability of Huntington muscle mitochondria against Ca2+ in R6/2 mice.. Ann Neurol.

[25] Rogers TB, Inesi G, Wade R, Lederer WJ (1995). Use of thapsigargin to study Ca2+ homeostasis in cardiac cells.. Biosci Rep.

[26] Kruman II, Mattson MP (1999). Pivotal role of mitochondrial calcium uptake in neural cell apoptosis and necrosis.. J Neurochem.

[27] Nicholls DG, Budd SL (2000). Mitochondria and neuronal survival.. Physiol Rev.

[28] Geisler S, Holmstrom KM, Skujat D, Fiesel FC, Rothfuss OC (2010). PINK1/Parkin-mediated mitophagy is dependent on VDAC1 and p62/SQSTM1.. Nat Cell Biol.

[29] Ramakrishnan M, Jensen PH, Marsh D (2003). Alpha-synuclein association with phosphatidylglycerol probed by lipid spin labels.. Biochemistry.

[30] Koshkin V, Greenberg ML (2000). Oxidative phosphorylation in cardiolipin-lacking yeast mitochondria.. Biochem J.

[31] Renvoise B, Parker RL, Yang D, Bakowska JC, Hurley JH (2010). SPG20 Protein Spartin is Recruited to Midbodies by ESCRT-III Protein Ist1 and Participates in Cytokinesis.. Mol Biol Cell.

[32] Chen JS, Greenberg AS, Wang SM (2002). Oleic acid-induced PKC isozyme translocation in RAW 264.7 macrophages.. J Cell Biochem.

[33] Cole NB, Murphy DD, Grider T, Rueter S, Brasaemle D (2002). Lipid droplet binding and oligomerization properties of the Parkinson's disease protein alpha-synuclein.. J Biol Chem.

[34] Majumder PK, Pandey P, Sun X, Cheng K, Datta R (2000). Mitochondrial translocation of protein kinase C delta in phorbol ester-induced cytochrome c release and apoptosis.. J Biol Chem.

[35] Shavali S, Brown-Borg HM, Ebadi M, Porter J (2008). Mitochondrial localization of alpha-synuclein protein in alpha-synuclein overexpressing cells.. Neurosci Lett.

[36] Li WW, Yang R, Guo JC, Ren HM, Zha XL (2007). Localization of alpha-synuclein to mitochondria within midbrain of mice.. Neuroreport.

[37] Wolins NE, Quaynor BK, Skinner JR, Schoenfish MJ, Tzekov A (2005). S3-12, Adipophilin, and TIP47 package lipid in adipocytes.. J Biol Chem.

[38] Duchen MR (2000). Mitochondria and calcium: from cell signalling to cell death.. J Physiol.

[39] Duchen MR (2004). Mitochondria in health and disease: perspectives on a new mitochondrial biology.. Mol Aspects Med.

[40] Milakovic T, Quintanilla RA, Johnson GV (2006). Mutant huntingtin expression induces mitochondrial calcium handling defects in clonal striatal cells: functional consequences.. J Biol Chem.

[41] Olichon A, Emorine LJ, Descoins E, Pelloquin L, Brichese L (2002). The human dynamin-related protein OPA1 is anchored to the mitochondrial inner membrane facing the inter-membrane space.. FEBS Lett.

[42] Dowler S, Currie RA, Campbell DG, Deak M, Kular G (2000). Identification of pleckstrin-homology-domain-containing proteins with novel phosphoinositide-binding specificities.. Biochem J.

[43] Farkas DL, Wei MD, Febbroriello P, Carson JH, Loew LM (1989). Simultaneous imaging of cell and mitochondrial membrane potentials.. Biophys J.

[44] Zhao K, Zhao GM, Wu D, Soong Y, Birk AV (2004). Cell-permeable peptide antioxidants targeted to inner mitochondrial membrane inhibit mitochondrial swelling, oxidative cell death, and reperfusion injury.. J Biol Chem.

[45] Joshi DC, Bakowska JC (2011). Determination of mitochondrial membrane potential and reactive oxygen species in live rat cortical neurons. http://www.jove.com/details.stp?id=2704 doi: 10.3791/2704.. J Vis Exp.

[46] Kerbiriou M, Le Drevo MA, Ferec C, Trouve P (2007). Coupling cystic fibrosis to endoplasmic reticulum stress: Differential role of Grp78 and ATF6.. Biochim Biophys Acta.

[47] Mehta B, Begum G, Joshi NB, Joshi PG (2008). Nitric oxide-mediated modulation of synaptic activity by astrocytic P2Y receptors.. J Gen Physiol.

[48] Wu Z, Puigserver P, Andersson U, Zhang C, Adelmant G (1999). Mechanisms controlling mitochondrial biogenesis and respiration through the thermogenic coactivator PGC-1.. Cell.

[49] Marks JD, Boriboun C, Wang J (2005). Mitochondrial nitric oxide mediates decreased vulnerability of hippocampal neurons from immature animals to NMDA.. J Neurosci.

[50] Aromolaran AS, Zima AV, Blatter LA (2007). Role of glycolytically generated ATP for CaMKII-mediated regulation of intracellular Ca2+ signaling in bovine vascular endothelial cells.. Am J Physiol Cell Physiol.

[51] Cui L, Jeong H, Borovecki F, Parkhurst CN, Tanese N (2006). Transcriptional repression of PGC-1alpha by mutant huntingtin leads to mitochondrial dysfunction and neurodegeneration.. Cell.

[52] Tsien RY (1981). A non-disruptive technique for loading calcium buffers and indicators into cells.. Nature.

[53] Hajnoczky G, Robb-Gaspers LD, Seitz MB, Thomas AP (1995). Decoding of cytosolic calcium oscillations in the mitochondria.. Cell.

[54] Manfredi G, Yang L, Gajewski CD, Mattiazzi M (2002). Measurements of ATP in mammalian cells.. Methods.

