# Supplementary figures and images for: SPG20 Protein Spartin Associates with Cardiolipin via Its Plant-Related Senescence Domain and Regulates Mitochondrial Ca2+ Homeostasis
Source: PLoS One. 2011 Apr 29;6(4):e19290. doi: 10.1371/journal.pone.0019290 (PMC3084803; doi:10.1371/journal.pone.0019290)

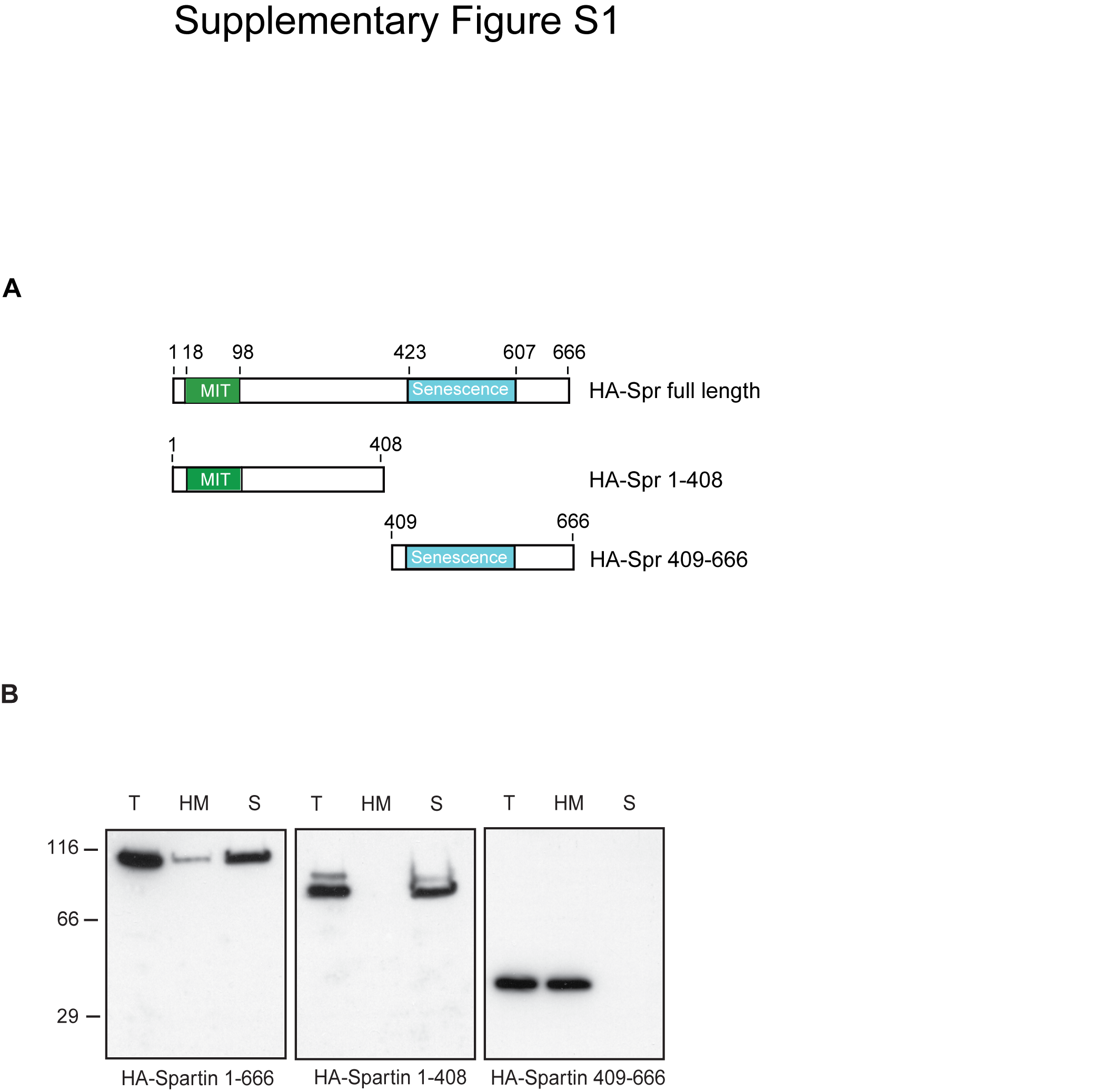

Supplement: Figure S1 — The association of spartin with mitochondria using fractionation. (A) Schematic diagrams of HA-tagged full-length spartin and deletion constructs encompassing microtubule interacting and trafficking (MIT) and/or plant-related senescence domain studied in a mitochondrial fraction. Numbers represent the amino acid residues, showing the boundaries of MIT and plant-related senescence domain. (B) SK-N-SH cells were transfected with indicated constructs of spartin, and the post-nuclear total homogenates (T) were fractionated into soluble (S) and mitochondria-enriched heavy-membrane fractions (HM) and immunoblotted with anti-HA antibodies. Sizes of protein standards are indicated to the left in kDa. (TIF) [file pone.0019290.s001.tif]

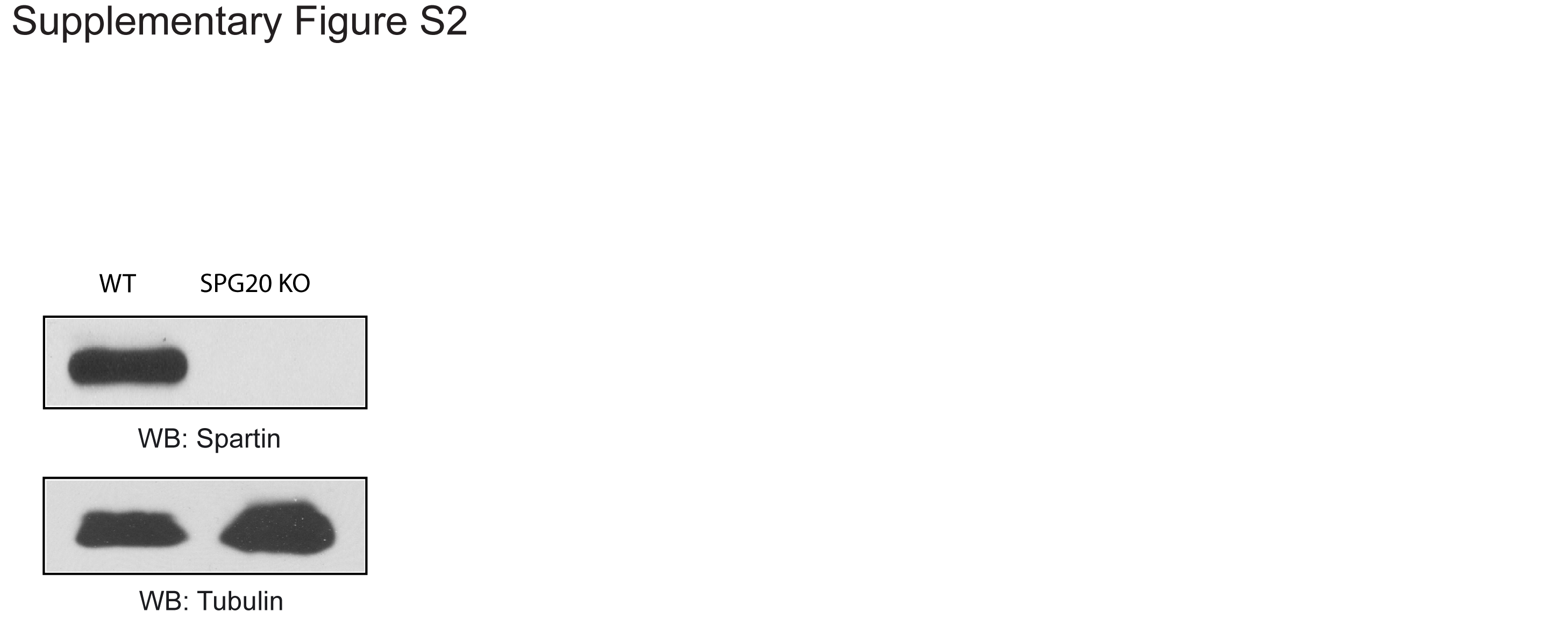

Supplement: Figure S2 — Analysis of expression of spartin in brain tissue from WT and Spg20 KO mice. Brain tissues from wild type (WT) and Spg20 KO mice were homogenized and immunoblotted with anti-spartin (upper panel) and β- tubulin antibodies (lower panel). (TIF) [file pone.0019290.s002.tif]

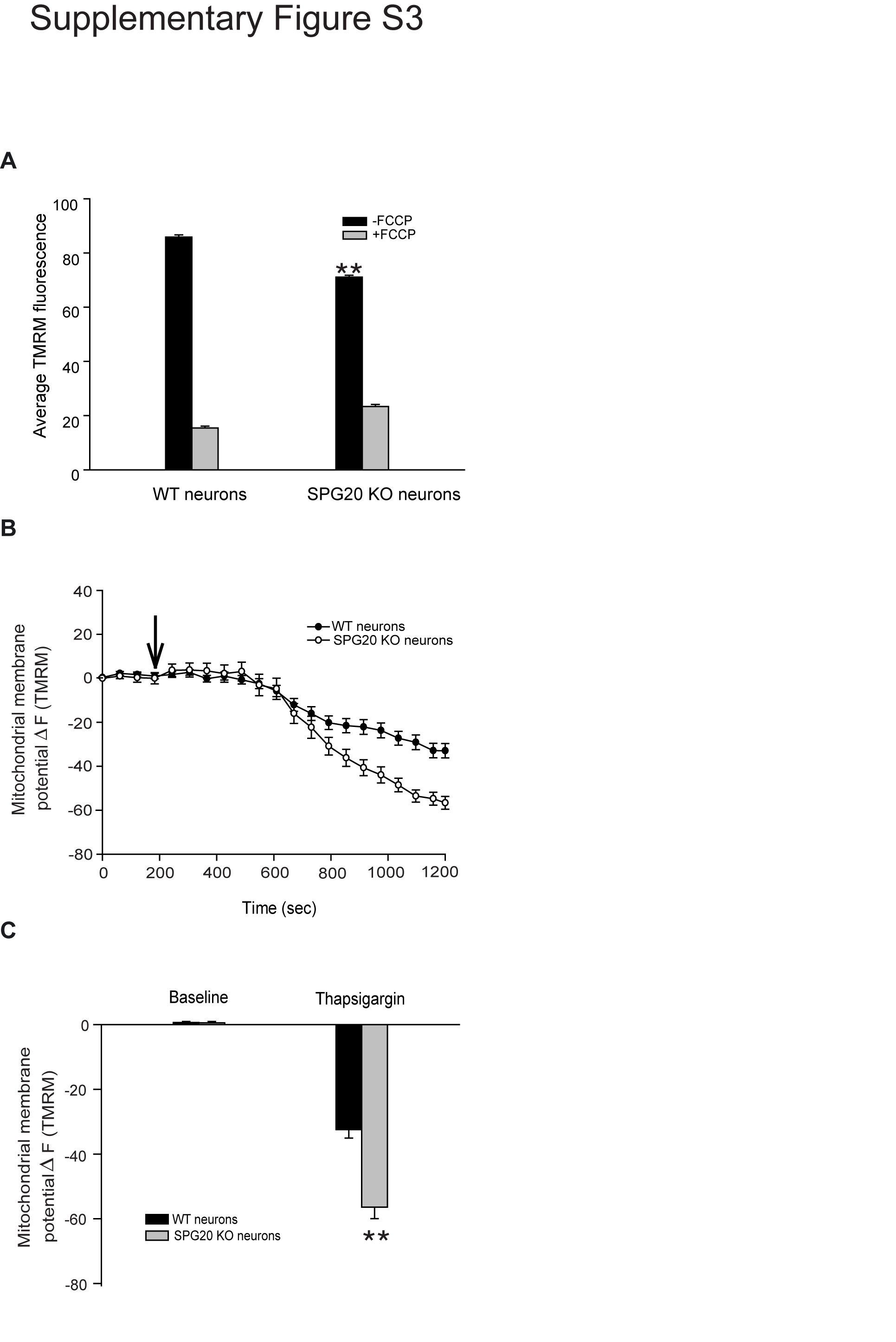

Supplement: Figure S3 — Depolarization of mitochondrial membrane potential in cultured primary cortical neurons derived from Spg20 KO mice. (A) Average pixel fluorescence intensity of TMRM from randomly selected mitochondrial regions in WT and Spg20 KO primary cortical neurons. Neurons were treated or not treated with the mitochondrial uncoupler, FCCP. (B) Changes in TMRM fluorescence intensity before and after treatment with 1 µM thapsigargin (indicated by arrow) in WT (black circle) and Spg20 mutant (white circle) neurons. (C) Bar graphs showing the relative fluorescence changes of TMRM representing the levels of mitochondrial membrane potential. Analysis was carried out in WT (black bars) or Spg20 mutant (gray bars) neurons at baseline (before stimulation with thapsigargin) and at 1200 sec after taking the first image. The data represent mean ± S.E.M in 75 neurons from three independent experiments (**p<0.001). (TIF) [file pone.0019290.s003.tif]

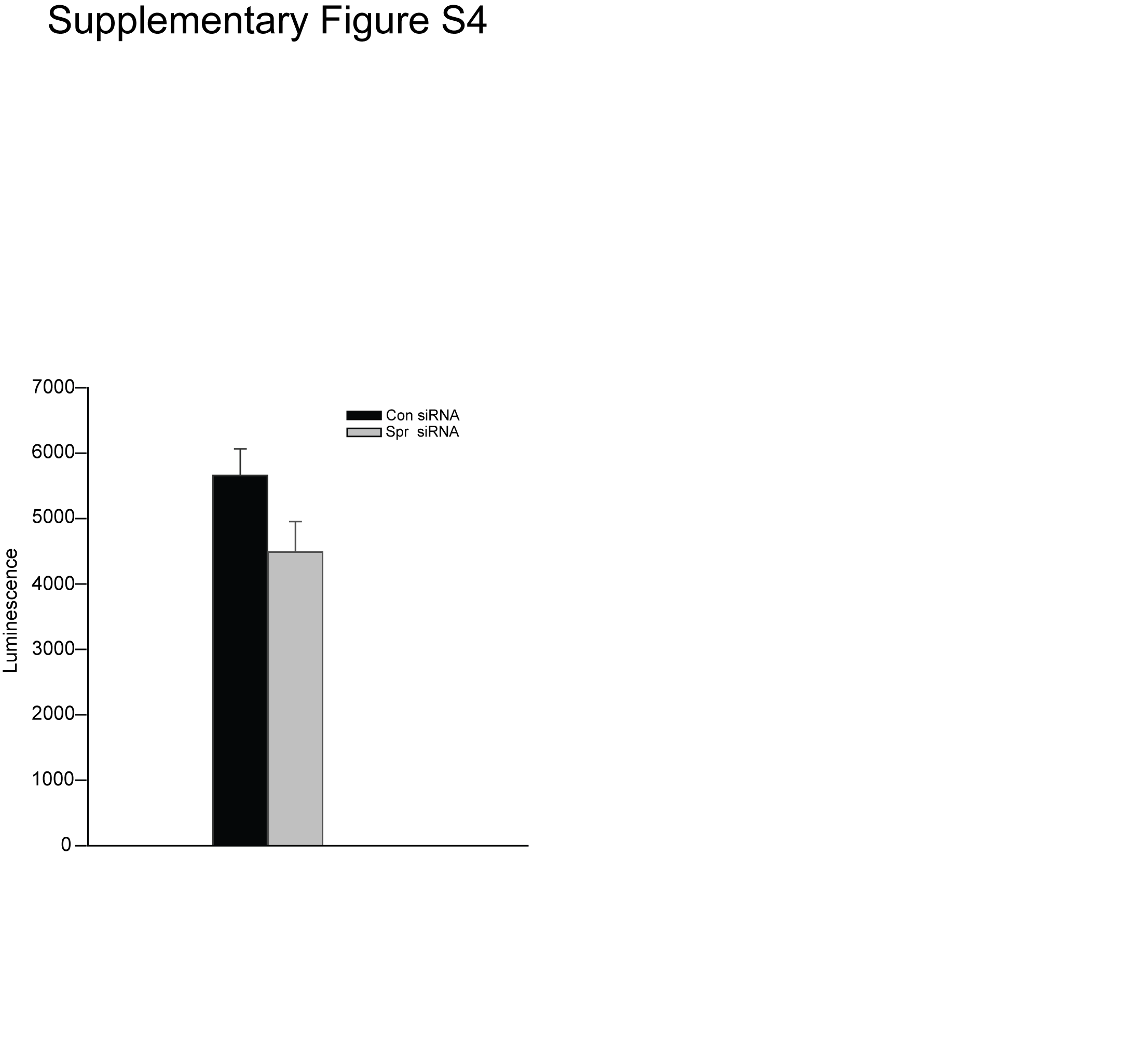

Supplement: Figure S4 — The levels of ATP in SK-N-SH cells treated with control or spartin siRNA. Cells were treated with siRNA for 48 hrs and ATP levels were measured using ATPlite luminescence assay kit (PerkinElmer) according to the manufacturer's protocol. Data represent mean ± SEM luminescence in triplicate treatment groups. (TIF) [file pone.0019290.s004.tif]

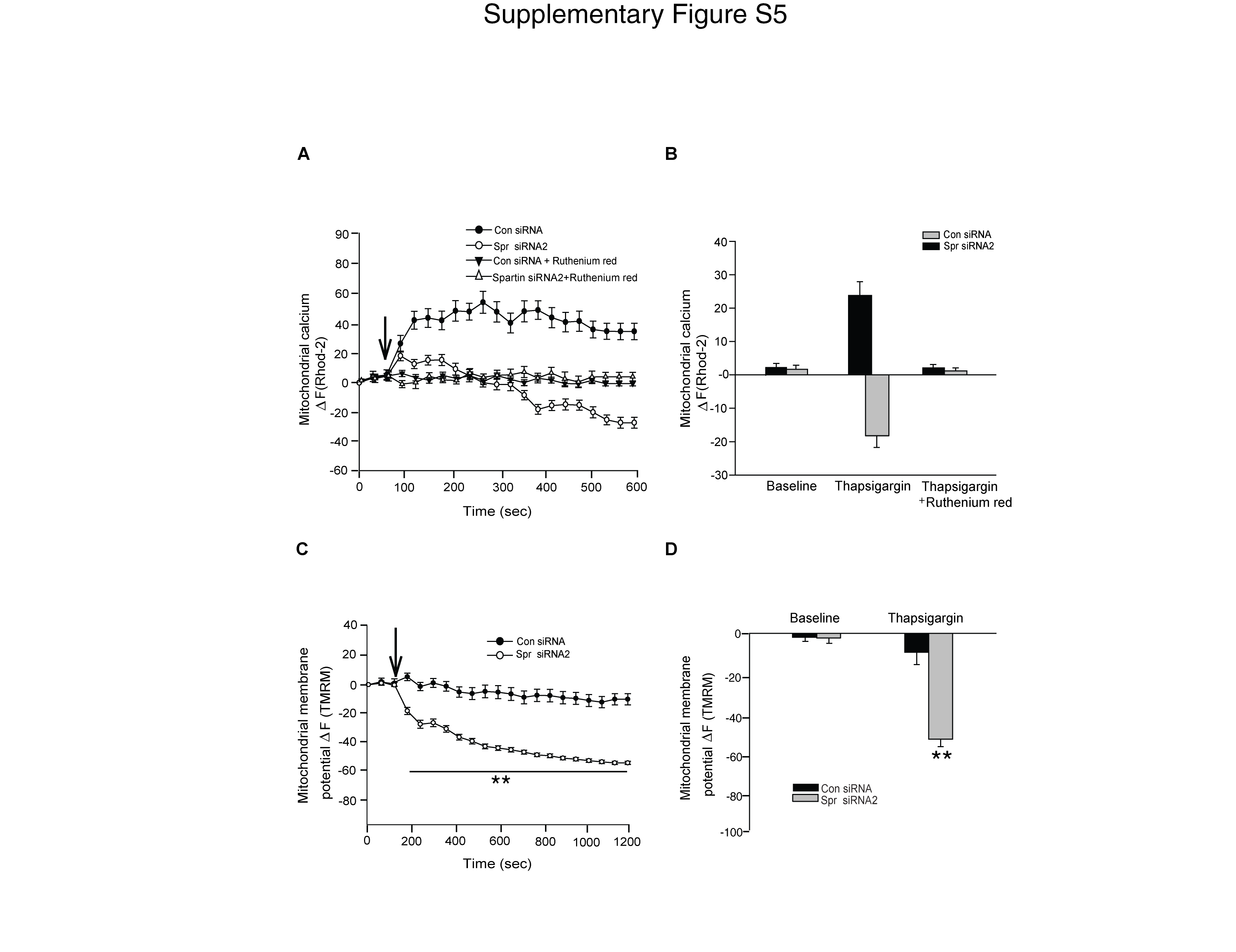

Supplement: Figure S5 — High intracellular Ca2+ levels cause mitochondrial dysfunction in spartin depleted SK-N-SH cells. (A) Changes in Rhod-2 fluorescence intensities (ΔF) upon 1 µM thapsigargin exposure in control siRNA (black circle) and spartin (white circle) siRNA2-treated cells. Fluorescence changes of Rhod-2 intensities were also measured in siRNA treated cells in the presence of mitochondrial Ca2+ uniporter blocker, Ruthenium red, prior to their stimulation with thapsigargin. Control siRNA (black triangles) and spartin siRNA (white triangles) depict changes in Rhod-2 fluorescence intensity upon thapsigargin exposure in cells treated with Ruthenium red. (B) The bar graph shows quantification of relative changes in Rhod-2 fluorescence intensity indicating the mitochondrial Ca2+ levels. Analysis was performed at baseline (before thapsigargin treatment), at 600 sec after the start of the experiment in control (black bars) and spartin siRNA2 (grey bars)-treated cells. Treatment groups are indicated on the X-axis. The data represent mean± S.E.M in 80 cells from two different experiments (**p<0.001). (C) Changes in TMRM fluorescence intensity (ΔF) upon 1 µM thapsigargin treatment (indicated by the arrow) in control (black circles) and spartin siRNA2 (white circles)-treated cells. (D) Bar graph representing the quantification of relative fluorescence changes of TMRM (ΔF) at baseline (before thapsigargin treatment) and at 600 sec after taking the first image in control (black bars) and spartin siRNA2 (grey bars) treated cells. The data represent mean ±S.E.M in 100 cells from three independent experiments (**p<0.001). (TIF) [file pone.0019290.s005.tif]
