# Supplementary material for: SPG20 Protein Spartin Associates with Cardiolipin via Its Plant-Related Senescence Domain and Regulates Mitochondrial Ca2+ Homeostasis
Source: PLoS One. 2011 Apr 29;6(4):e19290. doi: 10.1371/journal.pone.0019290 (PMC3084803; doi:10.1371/journal.pone.0019290)
Supplement: Text S1 — (DOC) [file pone.0019290.s006.doc]

**Supplementary Information**

**Animals and Neuronal Cultures**

The *Spg20* KO mice were generated as previously reported [1] and they were a generous gift of Dr. Blackstone. All animal experiments were approved by the ethics committee for Institutional Animal Care and Use Committee at Loyola University Chicago in accordance with the National Institutes of Health “*Guide for the Care and Use of Laboratory Animals*” (approval number IACUC#2010045). The preparation of murine cortical neurons was done as described previously [2].

**Measurement of Mitochondrial Ca^2+^ in the presence of Ruthenium red**

Mitochondrial uniporter dependent Ca^2+^ uptake was monitored in the presence of Ruthenium red, a potent inhibitor of mitochondrial Ca^2+^ uniporter. Briefly SK-N-SH cells were loaded with 5 µM of reduced Rhod-2 AM for 6 hrs. Then, the cells were washed three times with TB and incubated with 10 µM Ruthenium red in TB for 1 hr before thapsigargin treatment. Rhod-2 fluorescence was monitored in the presence or absence of thapsigargin as described in Material and Methods.

**Measurement of Mitochondrial Membrane Potential in Murine Cortical Neurons**

Mitochondrial membrane potential in primary cortical neurons was assessed by tetramethylrhodamine methyl ester (TMRM). Neuronal cultures on 8^th^ day in vitro (DIV) were washed 3 times with TB and incubated with TMRM (20 nM) in TB for 45 min at room temperature. The same concentration of TMRM was present throughout the experiment. Fluorescence imaging was performed using confocal microscopy (LSM 510). Images from randomly selected fields were collected by using a 40X water immersion objective at 514/570 nm excitation/emission with an argon laser at 1% transmission and 256 x 256 resolution. The fluorescence images were collected for 1 sec at an interval of 59 sec at axial resolution of 3.0 µm and a pixel depth of 12 bits. The change in TMRM fluorescence intensity was calculated using regions of interest (ROIs) similarly as in SK-N-SH cells. Mitochondrial localization of TMRM was confirmed using the mitochondrial uncoupler FCCP, which eliminates the TMRM fluorescence from mitochondria by collapsing the mitochondrial membrane potential.

**References**

1. Benoit Renvoise, Joanna Bakowska, Craig Blackstone (2010) Role of the ESCORT-III

Protein Ist1 in the Pathogenesis of Troyer Syndrome (SPG20). Abstract 51. American

Society for Biochemistry and Molecular Biology Conference.

2. Soderblom C, Stadler J, Jupille H, Blackstone C, Shupliakov O (2010) Targeted

disruption of the Mast syndrome gene SPG21 in mice impairs hind limb function and alters

axon branching in cultured cortical neurons. Neurogenetics. 11:369-78.
